# The Forensic Supplement to the interRAI Mental Health Assessment Instrument: Evaluation and Validation of the Problem Behavior Scale

**DOI:** 10.3389/fpsyt.2021.769034

**Published:** 2021-12-13

**Authors:** Howard E. Barbaree, Krista Mathias, Brant E. Fries, Greg P. Brown, Shannon L. Stewart, Elke Ham, John P. Hirdes

**Affiliations:** ^1^Department of Psychiatry, University of Toronto, Toronto, ON, Canada; ^2^Waypoint Centre for Mental Health Care, Midland, ON, Canada; ^3^interRAI Fellow, Saskatoon, SK, Canada; ^4^Institute of Gerontology, University of Michigan, Ann Arbor, MI, United States; ^5^School of Public Health, University of Michigan, Ann Arbor, MI, United States; ^6^Department of Criminal Justice, Nipissing University, North Bay, ON, Canada; ^7^Applied Psychology, Faculty of Education, Western University, London, ON, Canada; ^8^Department of Health Studies and Gerontology, University of Waterloo, Waterloo, ON, Canada

**Keywords:** risk assessment, forensic mental health, restraints, seclusion, coercive interventions, control procedures, patient safety

## Abstract

**Background:** Numerous validation studies support the use of the interRAI Mental Health (MH) assessment system for inpatient mental health assessment, triage, treatment planning, and outcome measurement. However, there have been suggestions that the interRAI MH does not include sufficient content relevant to forensic mental health. We address this potential deficiency through the development of a Forensic Supplement (FS) to the interRAI MH system. Using three forensic risk assessment instruments (PCL-R; HCR-20; VRAG) that had a record of independent cross validation in the forensic literature, we identified forensic content domains that were missing in the interRAI MH. We then independently developed items to provide forensic coverage. The resulting FS is a single-page, 19-item supplementary document that can be scored along with the interRAI MH, adding approximately 10–15 min to administration time.

We constructed the Problem Behavior Scale (PBS) using 11 items from the interRAI MH and FS. The Developmental Sample, 168 forensic mental health inpatients from two large mental health specialty hospitals, was assessed with both an earlier version of the interRAI MH and FS. This sample also provided us access to scores on the PCL-R, the HCR-20 and the VRAG. To validate our initial findings, we sought additional samples where scoring of the interRAI MH and the FS had been done. The first, the Forensic Sample (*N* = 587), consisted of forensic inpatients in other mental health units/hospitals. The second, the Correctional Sample (*N* = 618) was a random, representative sample of inmates in prisons, and the third, the Youth Sample (*N* = 90) comprised a group of youth in police custody.

**Results:** The PBS ranged from 0 to 11, was positively skewed with most scores below 3, and had good internal consistency (Cronbach's Alpha = 0.80). In a test of concurrent validity, correlations between PBS scores and forensic risk scores were moderate to high (i.e., r with PCL-R Factor two of 0.317; with HCR-20 Clinical of 0.46; and with HCR-20 Risk of 0.39). In a test of convergent validity, we used Binary Logistic Regression to demonstrate that the PBS was related to three negative patient experiences (recent verbal abuse, use of a seclusion room, and failure to attain an unaccompanied leave). For each of these three samples, we conducted the same convergent validity statistical analyses as we had for the Developmental Sample and the earlier findings were replicated. Finally, we examined the relationship between PBS scores and care planning triggers, part of the interRAI systems Clinical Assessment Protocols (CAPs). In all three validity samples, the PBS was significantly related to the following CAPs being triggered: Harm to Others, Interpersonal Conflict, Traumatic Life Events, and Control Interventions. These additional validations generalize our findings across age groups (adult, youth) and across health care and correctional settings.

**Conclusions:** The FS improves the interRAI MH's ability to identify risk for negative patient experiences and assess clinical needs in hospitalized/incarcerated forensic patients. These results generalize across age groups and across health care and correctional settings.

## Background

The most important problem faced by mental health professionals in a forensic inpatient environment is the ever-present threat to personal safety. Interpersonal violence by psychotic or personality-disordered patients necessitates staff use of coercive interventions to prevent serious injuries for both staff and patients. Therefore, comprehensive assessments of forensic patients must include an appraisal of their risk for violence while in hospital and their likely need for coercive intervention. The interRAI MH purports to be a comprehensive assessment for inpatient psychiatry. Nevertheless, there have been concerns raised in forensic mental health settings that, while the interRAI MH provides good coverage of content domains pertinent to general mental health issues, there are content domains relevant to forensic mental health that are not sufficiently covered. interRAI convened a task group to examine this issue and concluded that additional item content was needed to address forensic risk. Accordingly, as described in this article, we set out to devise a set of relevant items to be contained in a Forensic Supplement (FS) to the interRAI MH.

In addition, the present article describes our development of a scale, the Problem Behavior Scale (PBS), designed to predict negative outcomes experienced by forensic mental health patients in hospital. Specifically, the negative outcomes include interpersonal violence (perpetrated by the forensic inpatient) and the coercive interventions engaged in by staff to control the violent or potentially violent patient. Coercive interventions include: (1) environmental restraint, more commonly referred to as seclusion, (2) physical/mechanical restraint, and (3) chemical restraint (1). The issue of coercive interventions in mental health is fraught, with many perceiving these as an infringement of basic human rights and a threat to the therapeutic relationship, and others arguing that these interventions are necessary to ensure safety for staff and other patients. Our article does not directly address the appropriateness of coercive interventions. Rather, we attempt to develop an instrument to be incorporated into the interRAI MH system, to assess patients at admission to hospital to predict their need for coercive interventions. With a risk framework in place, it may be feasible to employ appropriate early interventions and de-escalation strategies to prevent the use of coercive interventions in some instances.

The article describes our study with two parts. In the first, we use a sample of forensic mental health patients to develop the PBS, including a preliminary evaluation of its reliability and validity. In the second, we sought to cross-validate this instrument with a second larger sample of forensic mental health patients and, using additional samples, to generalize the findings to other forensic settings, specifically adult prisons, and youth custody settings. For the prison sample, we analyzed data originally collected in a large study of prison inmates in Michigan (2). For the youth sample, we used data originally collected in a study of youth detained in Ontario, Canada (3).

### Patient and Staff Safety in Inpatient Mental Health

A systematic review of literature (4) on patient safety in mental health inpatient settings found 364 high-quality articles, including publications from over 31 countries, and involving over 150,000 inpatients. These studies focused on ten aspects of patient safety, and the top two concerns were interpersonal violence (116 articles) and coercive interventions (98 articles). When patients engage in violent behavior on a psychiatric ward, staff are required to control that behavior, and to reduce risk to other patients and staff. Coercive control techniques involve the use of physical restraints or seclusion and are used to prevent the individual from further violence. Coercive control techniques can be used effectively to control violence, but they have negative side effects for patients and staff. Patients in long-term restraints can suffer serious health effects including embolism leading to death. According to the Substance Abuse and Mental Health Services Administration (5), between 50 and 150 patients die each year in the US. Patients in seclusion can suffer long term, serious, and permanent psychological effects. Staff restraining or secluding a violent patient are also at risk of serious physical harm.

A meta-analysis of the world's literature (6) estimated the prevalence of violence among psychiatric inpatients. Their study included 122 surveys of psychiatric units around the world, including 12 countries and a total of 69,249 patients. The hospital units included acute care, forensic, and mixed units in mental health specialty hospitals. They estimated that 32.4% of psychiatric inpatients had been violent at least once while in hospital. They report that prevalence is much higher among forensic inpatients (47.7%) than acute care patients (26.2%) or general psychiatric patients (22.1%). These differences were even more significant when they compared incidence rates. Forensic units had an incident rate per 100 admissions of 406, while rates on acute care units (49) and general psychiatric units (39) were much lower.

A systematic review and meta-analysis (7) examined the use of risk assessment instruments used to predict violence while detained in forensic psychiatric hospitals. It identified the nine instruments most frequently used to assess violence risk, then conducted a systematic search of five international databases to identify studies examining the predictive accuracy of those tools in forensic inpatient settings. The authors identified risk assessment instruments designed for the prediction of short-term (within 24 h) risk for violence, and those designed for a longer-term prediction (i.e., weeks, months). This meta-analysis included data on 78 individual samples involving a total of 6,840 patients. The median AUC value was higher for short-term tools (AUC of 0.83) compared with longer-term tools (AUC of 0.68). The short-term tools were the *Broset Violence Checklist* (BVC) (8) and the *Dynamic Appraisal of Situational Aggression* (DASA) (9). Most samples assessed the performance of the *HCR-20* (10) (27 studies) and the *PCL-R* (11) (10 studies). These two instruments used for long term prediction performed moderately for the prediction of inpatient violence with median AUCs of 0.70 and 0.64, respectively.

### The interRAI MH

The interRAI MH is an assessment system for persons hospitalized with mental health issues, to improve care-planning by the identification of problems, risks, and strengths of the patient (12–14). It is completed by front line clinical staff at each patient's admission to hospital (within the first 3 days in hospital), discharge from hospital, and every quarter (every 3 months) for long-stay patients. Its 460 items cover a broad range of content areas relevant to health, mental health, hospital care, social supports, and use of support services. Scale scores derived from interRAI MH items measure critically important areas of functioning of the mental health inpatient. An earlier version of the interRAI MH [known as the Resident Assessment Instrument –Mental Health (RAI-MH)] was mandated by the Ministry of Health and Long-Term Care for use in all mental health inpatient facilities in the Province of Ontario and has been in use in mental health inpatient settings in Ontario since 1999, initially as a research instrument, but increasingly as part of clinical practice. The interRAI MH has numerous advantages, including that it has received extensive psychometric development and evaluation (12, 15–19).

An additional advantage of the interRAI MH is the availability of Clinical Assessment Protocols (20). These assist the clinician in planning effective clinical interventions to ensure improved outcomes for patients (21–23). interRAI has designed 21 CAPs, divided into five clinically meaningful categories (safety, social life, economic issues, autonomy, and health promotion). Once an interRAI MH assessment has been completed, CAPs are “triggered” for an individual patient based on algorithms operating on the assessment data. Many of the algorithms utilize one or more of 15 clinical scales (e.g., Cognitive Performance, Depression Rating, Mania, Positive Symptoms, etc.) derived from interRAI MH raw data. For example, the Harm to Others CAP (16) is triggered for a patient when the Risk of Harm to Others (RHO) Scale is above a set trigger level. The RHO scale is an empirically validated scale based on a history of violence, positive symptoms, insight, delusions, among other factors. Then, depending on which CAPs have been triggered, the associated guidelines provide the clinician with helpful resources to assist with care planning, identifying relevant evidence-based practices, advice to ensure safety, and recommendations for choosing appropriate outcome measures. Individual patients may have multiple CAPs triggered, resulting in a care plan with multiple goals and objectives. Incorporation of risk assessment capacity in the interRAI MH responds to the need for risk information upon admission to hospital since the interRAI MH assessment is done within 3 days of admission to hospital.

There are many items and scales contained in the interRAI MH that are importantly related to forensic mental health, including the Aggressive Behavior Scale (24) and the aforementioned RHO scale. Additional relevant CAPs include Criminal Activity, Interpersonal Conflict, and Control Interventions (20). Previous research has used the interRAI MH to examine the needs of forensic patients (25, 26), however, we pursued the development of the FS in response to expressed need for additional forensic risk content in this system. Hirdes et al. (14) reviewed the vast literature on the interRAI MH and argued convincingly that the instrument represented a comprehensive and integrated assessment of the mentally ill person in a variety of inpatient and outpatient hospital settings, as well as for individuals in the community being assessed for mental illness (e.g., courts, police). These authors provided strong evidence that the interRAI MH provided clinical teams with the required information to plan treatment and evaluate outcomes in a variety of clinical settings. There were only two areas where the instrument was thought to provide incomplete information, namely forensic and addictions settings. Hirdes et al. state that two supplements to the interRAI MH were being developed to provide additional information on problem severity, readiness for change, health symptoms associated with substance abuse and static and dynamic forensic risk factors. The present report is the second published study based on data collected using the Forensic Supplement (FS) (25).

### Use of the Term “Forensic”

In this article, we will be using the term “forensic” in two different ways: one general and one specific. Generally, a forensic patient is thought of a person with a mental disorder who is concurrently involved in the criminal justice system. For example, an outpatient receiving mental health services in the community may also be facing charges for a criminal offense. In the specific sense, different jurisdictions specify hospital patients as “forensic.” In Canada, hospital patients are designated as “Not Criminally Responsible” (NCR), or “Unfit to Stand Trial” or they are sent to hospital by the courts for forensic assessment. Our first two samples, the Developmental Sample and the Forensic Sample are “forensic” in the specific sense. They have been found NCR or Unfit by the courts and hospitalized for treatment or are detained in hospital by an order of the court for an assessment. Our other two samples, the Correctional Sample and the Youth Sample are “forensic” in the general sense.

## Methods

### Participants

The Developmental sample consisted of 168 mentally ill patients in medium and maximum secure forensic mental health inpatient units at two Ontario mental health facilities. The assessment was the RAI-MH (i.e., the earlier version of the interRAI MH) and a trial version of the Forensic Supplement with a detailed manual with instructions for scoring and training at each of the sites. These data also included participant scores on three risk assessment instruments that had a record of independent cross validation in forensic populations, namely: The Psychopathy Checklist-Revised (PCL-R) (11, 27, 28), The History, Clinical, Risk-20 (HCR-20) (10), and the Violence Risk Appraisal Guide (VRAG) (29). For the Developmental Sample, we collected: 109 VRAGs, 114 PCL-Rs, and 70 HCR-20s and used these data to develop the PBS and test for concurrent validity.

In addition, to provide for an evaluation of FS coding reliability, for 33 of these assessments, an independent second FS assessment was performed by a hospital Psychometrist based entirely on the hospital charts and records. These data were used to test FS item reliability.

Three separate samples were also used to validate the PBS, and to test its generalizability to other forensic populations. The first, the Forensic Sample, was a sample of 587 unique mental health patients across four forensic units/hospitals in Ontario. The second was a Correctional Sample consisting of a stratified, random, representative sample of mental health assessments in Michigan prisons (2). Prisoners were randomly sampled based on four strata: males in the general population, males in administrative segregation, males in special units, and females. A total of 618 incarcerated subjects were assessed using the interRAI Correctional Facilities Instrument which consisted of both interRAI MH and FS items. The final validity sample, our “Youth” sample, consisted of 90 youth between the ages of 16 and 18 in detention or custody in Ontario who consented to participate. This sample was a subset of a large sample (*N* = 755) of youth (Mean age = 16.75, SD = 0.81), 47% male, divided into three groups: inpatient and outpatient mental health, and in custody. The inpatient and outpatients were referred from 22 mental health agencies in Ontario, and the in-custody sample were referred from 10 secure custody sites across Ontario (3, 30). The interRAI instrument used for data collection with this sample was the interRAI Youth Justice Custodial Facilities Instrument (31), which is based on the interRAI Child and Youth Mental Health assessment system (32) and certain items have considerable overlap with items (with identical wording) in the interRAI MH.

### Research Ethics Approval

Research on the Developmental and the Forensic Samples was approved by the REBs at the following institutions: the University of Waterloo, Waypoint Center for Mental Health Care, The Center for Addiction and Mental Health.

Research on the Michigan correctional sample was approved by the institutional Review Boards of the University of Michigan, the Michigan Public Health Institute, and the Michigan Department of Community Health.

Ethics approval for the Youth Justice Sample was provided by the University of Toronto, the Center for Addiction and Mental Health, the University of Western Ontario, the Ministry of Children and Youth Services (MCYS) Judicial Review, MCYS's internal ethics review and two facilities requiring separate ethics protocols. To be deemed competent, youth were required to understand the foreseeable risks, potential benefits, as well as the consequences of participating in the research study.

### Procedure

We started our work examining the three risk assessment instruments – the PCL-R, HCR-20, and VRAG– to identify item domains that were missing from the interRAI MH. From the PCL-R, most of items contained in Factors 1-3 from the PCL-R's Four Factor Model [Hare (11), pg. 83] were missing. There were item domains missing from the HCR-20 and VRAG as well, including the development of antisociality, antisocial attitudes and failure on prior supervised release.

Then, we wrote items to capture forensic risk content according to the traditional interRAI item format paying particular attention to respecting the intellectual property rights of the original forensic instrument developers. The supplement itself was designed to add only one page to the current interRAI MH and an additional 5–10 min to the administration time.

A section on Mental State Indicators included: remorseless; impulsive; inappropriately blames others for problems; denies or minimizes harm done to others; and expressions supportive of criminal activity. A section on social relations included: manipulative; lacks empathy; and takes advantage of others. Other additions included: age at first police intervention for criminal activity; severity of crime; victims were women or children; use of a weapon; early behavior problems; and failure to comply with conditions of any release. Additional items included: issues relating to resources for discharge such as: understands and identifies sources of stress; enacts appropriate strategies; and unrealistic plans. With respect to juvenile delinquency, items added included: removed from home before age 18; and antisocial peer group.

### Analytic Approach

To develop the Problem Behavior Scale, we chose 11 items from the FS and interRAI MH that represented item domains figuring prominently on the three risk assessment instruments cited above (VRAG, PCL-R, and HCR-20). To ensure that each item contributed equally to the scale, all the multi-level items were recoded into dichotomies representing “Present “or “Not Present.” Then the item scores were added together to form a scale with total scale scores ranging from 0 to 11. We then conducted separate Binary Logistic Regression Analyses using the PBS scale to evaluate relations with patient experiences in the care setting (verbal abuse, use of restraints, seclusion, etc.) For each the Wald Statistic was tested for significance. In addition, we conducted a Receiver Operating Characteristic Analysis (ROC), again using the PBS score to predict hospital experiences. AUCs are reported in **Table 4**.

### Computer Programs

Analyses of the Developmental and Youth Samples were performed using IBM SPSS Statistics Package, Version 25; analysis of the Correctional sample data was performed using SAS, Version 9.4 and of the Forensic Sample, SAS Version 9.3.

## Results

### Participants

#### Developmental Sample

The mean age of the sample was 41 years of age. There were 146 (96%) males; 146 (92%) were English speaking; 104 (88%) were never married. With respect to education, 22 (14%) had grade eight or less, 76 (49%) had some high school, 35 (22%) had graduated high school, and 16 (10%) had at least some postsecondary education. For the remainder, education was minimal or not known.

#### Forensic Sample

The mean age of the sample was 41 years of age. There were 482 (83%) males and 100 (17%) females 0.540 (93%) were English speaking 0.429 (73%) were never married, 74 (13%) were married or had a live-in partner, and the remainder were separated/divorced/widowed. With respect to education: 36 (6%) had no schooling, 62 (11%) had grade eight or less, 127 (22%) had some high school, 114 (20%) had graduated high school, and 99 (17%) had at least some postsecondary education. For the remainder, education level was not known.

#### Correctional Sample

A total of 618 inmate participants were recruited and interviewed. An additional 262 inmates were approached by correctional staff but either declined to be interviewed or refused to give consent. Two interviews were stopped halfway when it was deemed that the subject was incompetent to provide useful information. Of all prisoners, 78% were between the ages of 20 and 50 years; median and modal age was between 30 and 39 years 0.96% were male and half were black with only a slightly smaller % white. Over half the sample had less than a high school level education.

#### Youth Sample

The sample (*N* = 90) was an in-custody subset of a larger sample (*N* = 755) of youth aged 16–19 years of age, involved in criminal justice and mental health facilities in Ontario, Canada. The in-custody participants were referred from 10 secure custody sites across the Province of Ontario. The average (mean) age was 17.24 years (SD = 0.89) and 77% of the sample was male.

### Reliability of the Forensic Supplement and Problem Behavior Scale Items

#### FS Coding Reliability

Table 1 presents the results of the reliability study of the FS items. Items were divided into six categories listed in column one in the table. Both the mean category reliabilities and those of individual items were all acceptable, with many achieving excellent reliability. We used Cohen's categorization of Kappa's (33) that he suggests be interpreted as follows: values ≤ 0 as indicating no agreement, 0.01–0.20 as none to slight, 0.21–0.40 as fair, 0.41–0.60 as moderate, 0.61–0.80 as substantial, and 0.81–1.00 as excellent. Overall, using the smaller sample (*N* = 33), the 11 items that are utilized in the PBS (remorseless, impulsive, inappropriately blames others, denies, or minimizes harm, expressions supportive of criminal activity, manipulative, lacks empathy, takes advantage of others, inflated self worth, irritability, and anger) have a Cronbach's Alpha of 0.84. We view these measures of reliability as estimates of the lower bounds of overall interrater reliability, as one of the raters only had partial information (e.g., no access to direct observation, team meetings, etc.).

**Table 1:** Interrater reliability for items on the Forensic Supplement to the RAI=MH.

**Item category**	**# of items**	**Mean % agreement**	**Mean kappa**	**Nominal reliability**
Mental state indicators	5	67	0.55	4 moderate,1 substantial
Criminal involvement	7	82	0.56	3 moderate, 3 substantial, 1 excellent
Behavior	2	94	0.87	1 substantial, 1 excellent
Life events	1	81	0.67	1 substantial, 1 excellent
Social relations	4	83	0.63	2 moderate, 2 substantial
Resources for discharge	2	91	0.66	2 substantial

### Characteristics and Psychometrics of the PBS Scale

#### Concurrent Validity

Table 2 presents intercorrelations among the risk assessment instruments scores and correlations between the PBS score and risk assessment scores. The shaded area in the table presents the three intercorrelations among the risk assessment scores. These correlations were remarkably high and range between 0.52 and 0.76, as one would expect from three validated risk instruments. The top row of the table presents the correlations between the PBS score and the various risk assessment scores. These range from 0.26 to 0.46. These correlations are moderately high and reflect a reasonable-to-high degree of concurrent validity.

**Table 2:** Inter-correlations among risk instrument scores and correlations between PBS scores and each risk score.

					**HCR-20**	**HCR-20**
	**PBS**	**VRAG**	**PCL-R**	**HCR-20**	**Clinical**	**Risk**
PBS	XXX	0.278^**^	0.260^**^	0.370^**^	0.461^**^	0.386^**^
VRAG		XXX	0.764^***^	0.545^***^		
PCL-R			XXX	0.517^***^		
HCR-20				XXX		

** *p < 0.01*.

*** *p < 0.001*.

#### Endorsement of PBS Items

**Figure 1:**
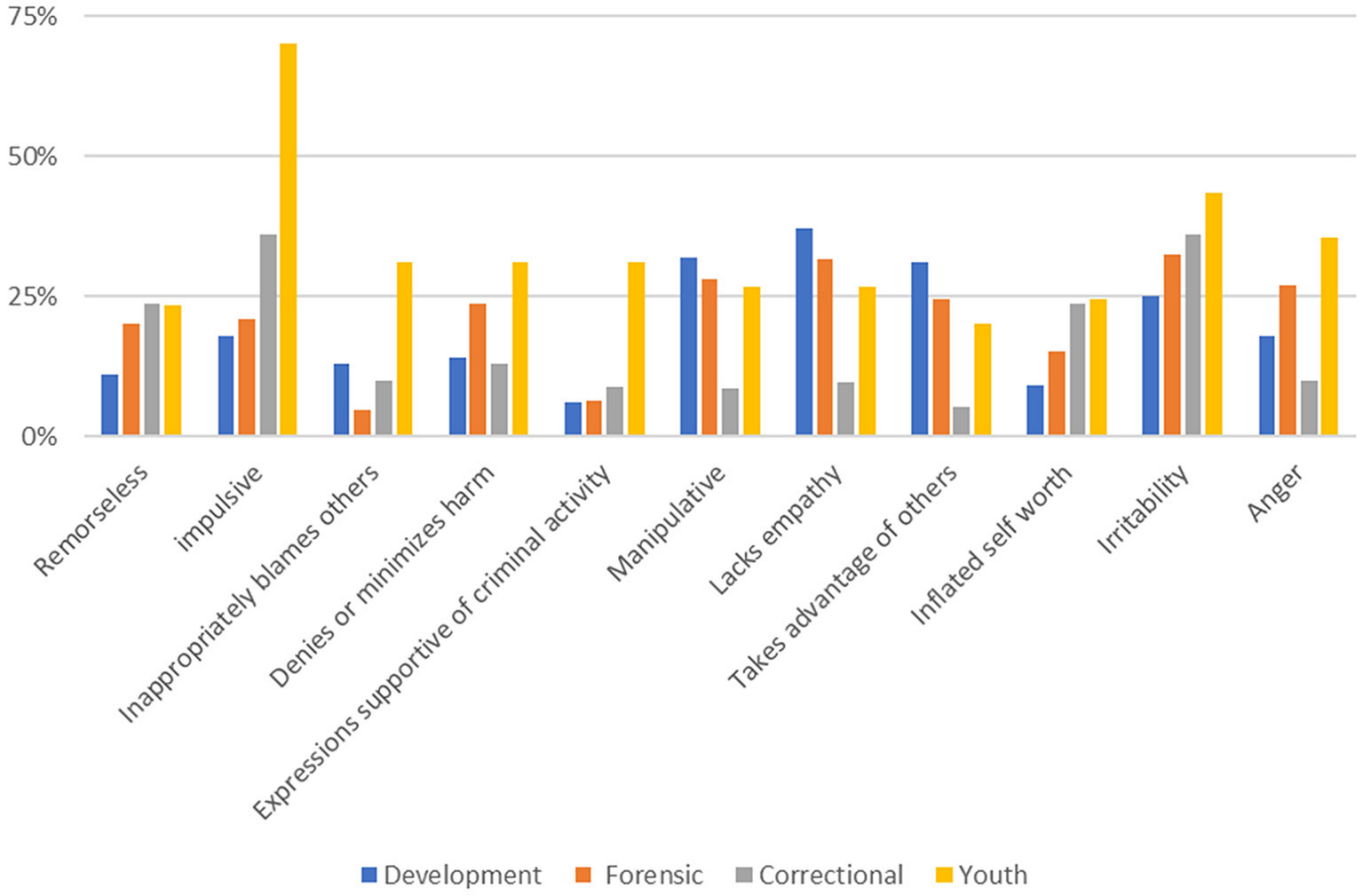
Rates of endorsement of PBS items in four study samples.

The rate of endorsement of PBS items is shown in Figure 1 and ranged from 6 to 70% over the 11 items. Rates in the Developmental and Forensic samples were similar. The rate of endorsement of the 11 items ranged from 5% (for “inappropriately blames others”) in the Developmental sample, to 33% (for “irritability”) in the Forensic sample. The other two samples showed a different pattern across these 11 items. For example, in the Correctional Sample, the rates for “manipulative,” “lacks empathy,” and “anger” were at least half those in the two hospital-based samples. Similarly, in the Youth Sample, the endorsement rate for “Impulsive” was seen over three times more frequently than in the hospital samples. Endorsements ranged from 5% for the item “takes advantage of others,” to 36% for the items “impulsive” and “irritability.” However, for the Youth Sample, rates of endorsement for these items were much higher. The lowest endorsement rate was 23% for the item “remorseless” and the highest endorsement rate is 70% for the item “impulsive.” At least in terms of item endorsement, the youth sample was different from the adult sample in having a higher rate of endorsement of PBS items. These differences were reflected as well in the overall scale values with the Youth sample demonstrating higher values overall, including a higher mean (3.6 compared with >2.3 for the other three samples, see ANOVA results below).

#### Distributional Properties

**Figure 2:**
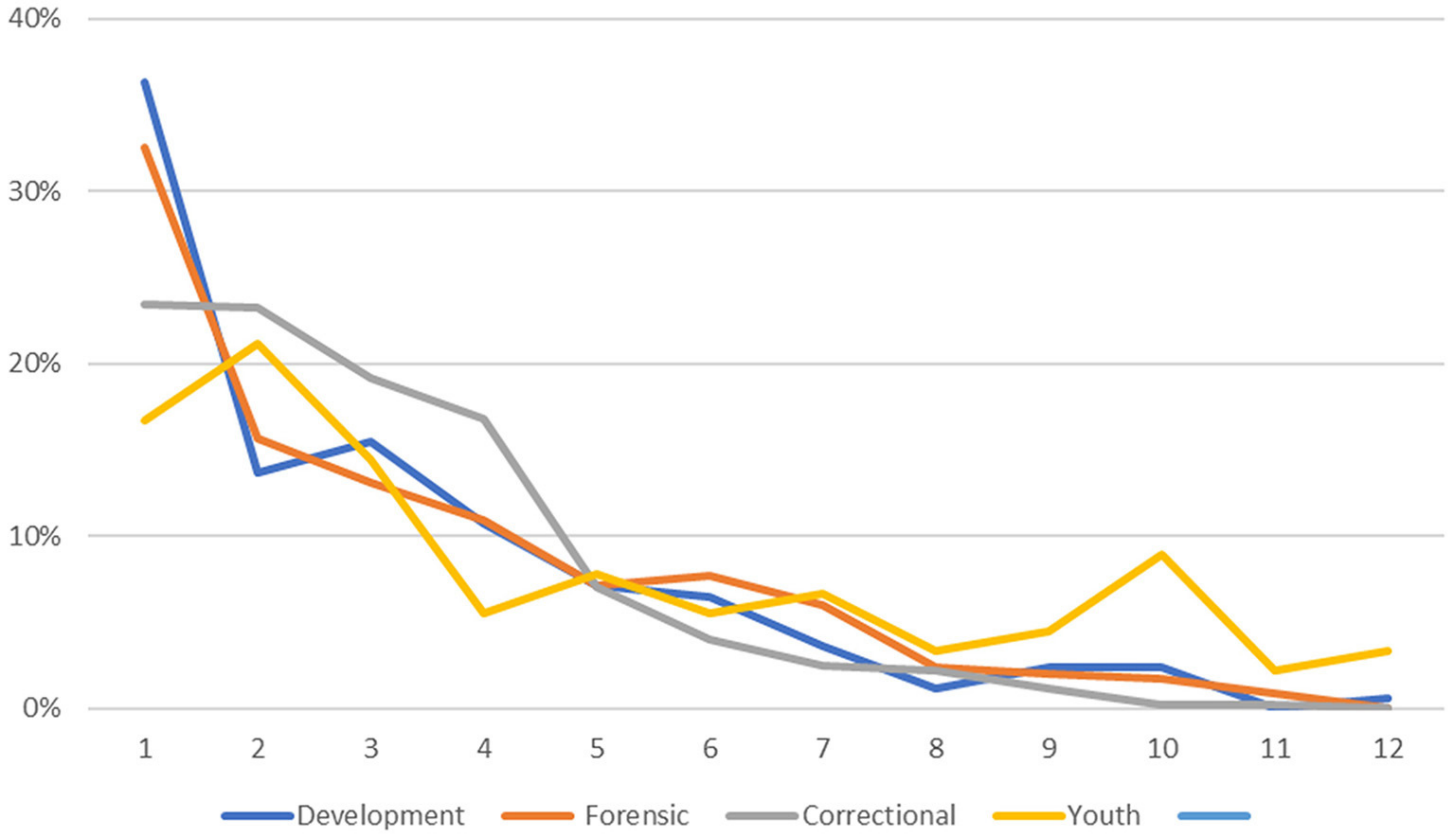
Frequency histogram of PBS score values for four study samples.

As can be seen from Figure 2, the distributions were positively skewed; for all four samples, the mean scores were numerically higher than the median scores. In the Developmental Sample, the 90th percentile was reached at score five of 11, the mean score was 2.15 and the median score was 1.90. Only 10% of the Developmental Sample had scores above five and the number of scores for each of the remaining scores decreased to score 11. The three validity samples, like the Developmental sample, had positively skewed distributions. For example, for the Forensic Sample, the 90th percentile was reached at score 6, and the mean was 2.34 and the median was 2.03. Similarly, for the Correctional Sample, the 90th percentile was reached at score five on the PBS, the mean score was 2.04 and the median was 1.18. However, the youth sample was somewhat different, in terms of the mean PBS score and in the shape of the distribution. The mean score was 3.63 and the median was 1.96. The distribution of scores did not reach the 90th percentile until the PBS score of 9. This sample distribution showed less Kurtosis than the other three distributions with more of the sample scoring higher numbers on the scale.

It is apparent that that the Youth Sample had higher PBS scores than the other samples. A One-way Analysis of Variance shows that the means differed significantly [Mean Scale Values: Developmental 2.20, Forensic 2.34, Correctional 2.00, Youth 3.60, F_(3, 1435)_ = 12.78, *p* < 001]. Tukey's Honestly Significant Difference (HSD) test found an HSD of 0.397 indicating that the mean PBS score of the youth group was significantly higher than the other means, and that there were no significant differences among the other three means.

#### Internal Consistency

We calculated the internal consistency of the PBS using Cronbach's Alpha, and these are presented in Table 3. For the Developmental sample, internal consistency was strong at 0.80. The three validation samples showed similar Alphas (0.81, 0.70, and 0.88 for the Forensic, Correctional, and Youth samples, respectively).

**Table 3:** Internal consistency of the problem behavior scale (PBS) items (Coefficient Alpha with item deleted).

**Samples**	**Developmental**	**Forensic**	**Correctional**	**Youth**
	**sample**	**sample**	**sample**	**sample**
Item				
Remorseless	0.78	0.80	0.68	0.86
impulsive	0.80	0.80	0.70	0.88
Inappropriately blames others	0.78	0.82	0.69	0.86
Denies or minimizes harm	0.77	0.79	0.66	0.86
Expressions supportive of criminal activity	0.79	0.81	0.69	0.87
Manipulative	0.78	0.79	0.66	0.87
Lacks empathy	0.78	0.79	0.65	0.86
Takes advantage of others	0.79	0.79	0.67	0.87
Inflated self worth	0.79	0.81	0.71	0.86
Irritability	0.80	0.80	0.72	0.87
Anger	0.80	0.80	0.69	0.87
Coefficient Alpha	0.80	0.81	0.70	0.88
			Average Alpha =	0.80

### Validation of the PBS

#### PBS Related to Negative Hospital Outcomes

Table 4 presents data and outcomes from our evaluation of the PBS's ability to predict negative outcomes in hospital. The first column in the table lists negative outcomes. We used binary logistic regression to analyze the relationship between PBS scores and various hospital outcomes. The results of the logistic regressions are tabulated in Table 4. In addition, we calculated a ROC analysis, and AUC values are presented for each finding in the text. In the Developmental sample, we found three outcomes where the PBS score predicted outcomes: “verbal abuse” (AUC = 0.79, CI 0.70–0.88, *p* < 0.0001, “seclusion” (AUC = 0.77, CI 0.63–0.91, *p* < 0.0001), and “unaccompanied leave” (AUC = 0.75, CI 0.66–0.84, *p* < 0.001). Note that higher PBS scores were associated with increased “verbal abuse” and the use of “seclusion,” whereas lower PBS scores were associated with increased unaccompanied leave). In terms of validation, “verbal abuse” was found to be significantly predicted in all three validity samples. In the Developmental sample, prediction of “physical and manual restraint” was not significant, but it was significantly predicted by the PBS in the Forensic Sample.

**Table 4:** Convergent validity of the Problem Behavior Scale (PBS).

**Negative outcomes**	**Outcome**			**Logistic regression**				**AUC analysis**		
	***N***	**%**	**B**	**SE**	**OR (CI 95%)**	**Wald**	***P***	**AUC**	**CI 95%**	***P***
Developmental sample	168									
Verbal abuse		8.3	0.34	0.1	1.41 (1.16–1.71)	12.07	<0.001	0.79	0.70–0.88	<0.0001
Seclusion		7.1	0.34	0.1	1.4 (1.15–1.73)	11	<0.001	0.77	0.63–0.91	<0.0001
Unaccompanied leave		18.5	−0.64	0.18	0.53 (0.37–0.75)	12.41	<0.001	0.75	0.66–0.84	<0.001
Physical/Manual Restraint		4.8	0.128	0.128	1.14 (0.88–1.46)	0.999	0.317	0.36	0.20–0.52	ns
Forensic Sample	587									
Verbal Abuse		15.0	2.24	0.22	9.40(6.09–14.53)	101.97	<0.0001	0.86	0.84–0.88	<0.0001
Seclusion		5.1	1.07	0.33	2.91(1.53–5.55)	10.52	<0.001	0.79	0.74–0.83	<0.0001
Unaccompanied leave		16.4	−0.72	0.2	0.48 (0.32–0.72)	12.62	<0.001	0.61	0.58–0.63	<0.0001
Physical /Manual Restraint		4.8	1.94	0.35	6.97 (3.53–13.76)	31.26	<0.0001	0.8	0.75–0.84	<0.0001
Correctional sample	618									
Intimidation		17.7	0.3378	0.0542	1.40 (1.26–1.55)	38.77	<0.0001	0.72	0.66–0.76	<0.0001
Verbal abuse		23.3	0.5586	0.06	1.75 (1.55–1.97)	84.2375	<0.0001	0.77	0.73–0.82	<0.0001
Resists care		4.2	0.4643	0.0872	1.59 (1.34–1.89)	28.32	<0.0001	0.76	0.65–0.87	<0.0001
Physical/manual restraint		1.2	0.25	0.16	1.28 (0.94–1.76)	2.42	ns	0.73	0.58–0.88	0.0033
Unit confinement		20.6	0.2	0.05	1.22 (1.10–1.34)	14.71	<0.001	0.63	0.57–0.68	<0.0001
Segregation		29.4	0.37	0.05	1.45 (1.31–1.60)	52.21	<0.0001	0.69	0.64–0.73	<0.0001
Youth sample	90									
Intimidation		41.1	0.17	0.07	1.19 (1.04–1.36)	6.45	<0.02	0.67	0.56–0.79	<0.01
Verbal abuse		45.6	0.94	0.19	2.55 (1.75–3.71)	23.57	<0.001	0.93	0.89–0.98	<0.0001
Resists care		10.0	0.28	0.11	1.33 (1.07–1.64)	6.85	<0.01	0.78	0.63–0.92	<0.0001

“Seclusion” was significantly predicted in the Developmental and Forensic Samples (AUC = 0.79, CI 0.74–0.83, *p* < 0.0001 and AUC = 0.77, CI 0.63–0.91, respectively). The equivalent negative outcomes in corrections, “confinement to unit” (AUC = 0.63, CI 0.57–0.68, *p* < 0.0001), and “segregation” (AUC = 0.69, CI 0.64–0.73, *p* < 0.0001) were significantly predicted in the Correctional Sample. The Youth Sample showed no replication of “seclusion,” however, in the Youth Sample, the PBS was predictive of “intimidation” (AUC = 0.67, CI 0.56–0.79, *p* < 0.01), “verbal abuse” (AUC = 0.93, CI 0.89–0.98, *p* < 0.0001) and “resists care” (AUC = 0.78, CI 0.63–0.92, *p* < 0.0001).

#### PBS Related to CAPS Triggered

Finally, Table 5 describes our statistical analyses and outcomes in our prediction of Clinical Assessment Protocols (CAPs) being triggered. We used binary logistic regression to examine the relationship between PBS scores and CAPs according to rules laid out for each CAP in the CAPs manual see Hirdes et al. (20). For all three validity samples, CAPs predicted by the PBS were “Harm to Others,” “Interpersonal Conflict,” “Traumatic Llife Events,” and “Control Interventions.” Adult participants in forensic mental health care in Ontario, in jail in Michigan, and youth in custody in Ontario, who score higher on the PBS were more likely to “trigger” the same four clinical assessment protocols.

**Table 5:** Relationship of problem behavior scale to RAI-MH clinical assessment protocols (CAPs).

**Validation samples**	**Clincal assessment protocols**	**Cap triggered**		**Logistic regression**				
		***N***	**%**	**B**	**SE**	**OR (CI 96%)**	**Wald**	***P***
Forensic Sample		587						
	harm to others		39.76	0.97	0.11	2.64 (2.132–3.268)	79.35	<0.0001
	interpersonal conflict		50.94	1.34	0.11	3.82 (3.081–4.741)	148.58	<0.0001
	traumatic life events		11.75	0.26	0.15	1.301 (0.975–1.737)	3.19	ns
	control interventions		18.23	0.94	0.12	2.561 (2.021–3.246)	60.48	<0.0001
Correctional Sample		618					
	Harm to others		43.12	0.48	0.06	1.62 (1.44–1.82)	69.50	<0.0001
	Interpersonal conflict		61.04	0.96	0.09	2.60 (2.18–3.11)	112.95	<0.0001
	Traumatic life events		39.93	0.17	0.05	1.18 (1.08–1.30)	13.96	0.0002
	Control interventions		29.37	0.37	0.05	1.45 (1.30–1.60)	47.97	<0.0001
Youth Sample		90						
	Harm to others		48.90	0.20	0.05	1.26 (1.09–1.45)	15.54	<0.0001
	Interpersonal conflict		72.20	0.30	0.09	1.42 (1.14–1.77)	11.17	<0.0001
	Traumatic life events		56.70	0.22	0.06	1.26 (1.08–1.46)	12.00	<0.001
	Control interventions		34.40	0.16	0.06	1.15 (1.01–1.32)	8.90	<0.001

## Discussion

The interRAI MH is a “comprehensive standardized instrument for evaluating the needs, strengths and preferences of adults with mental illness in in-patient psychiatric settings” [(34), p. 2–4]. It covers a broad range of content areas including mental health, health, hospital care and social supports. Completed by front-line clinical staff at admission, discharge and quarterly for longer-term patients, the RAI-MH provides for frequent assessment and monitoring of patients. In response to calls from forensic mental health settings to develop additional content domains that address the symptoms, behaviors and needs unique to these settings, the Forensic Supplement (FS) was developed in an Ontario Pilot Study. Based on and validated against other forensic risk assessment instruments, the FS is a short, 19-item instrument completed by front-line clinical staff. In a pilot study, the FS demonstrated an acceptable level of coding reliability.

Patient and staff safety is a special concern in mental health settings. Interpersonal violence and use of coercive interventions have been identified as the two most important safety concerns (4). These “negative outcomes” of mental health care can lead to serious physical injury for patients and staff and other negative health and psychological effects for patients stemming from the use of coercive interventions or seclusion. Combining items from the RAI-MH and the FS, a Problem Behavior Scale (PBS) was developed to assess patient risk for these negative outcomes while in a forensic mental health setting.

Comprised of eleven items, three from the RAI-MH and eight from the FS, the PBS demonstrated excellent overall scale reliability and moderately high correlation of risk scores with the VRAG, PCL-R and HCR-20 instruments, demonstrating good concurrent validity in a developmental sample. In validation of the PBS with the Forensic mental health sample, the Michigan State correctional validation sample and the Ontario Youth Custody validation sample, good internal consistency was demonstrated. The PBS significantly predicted negative outcomes in the Developmental Sample (verbal abuse, seclusion, unaccompanied leave), in the Forensic Sample (verbal abuse, seclusion, unaccompanied leave, physical/manual constraint), the Correctional Sample (intimidation, verbal abuse, resists care, unit confinement, segregation) and the Youth Sample (intimidation, verbal abuse, resists care). In addition, the PBS significantly predicted the triggering of four CAPS already included in the RAI-MH, including Harms to Others, Interpersonal Conflict, Control Interventions, and Traumatic Life Events.

The PBS demonstrated strong convergent validity for negative outcomes across a variety of forensic settings, including mental health, a state prison population, and youth in secure custody. Among the Youth in Custody, the endorsement of PBS items, internal consistency, and distribution of scores on the PBS was especially strong, a consequence of the historical significant decline in the number of youths sentenced to secure custody in Ontario, with a relatively small number of predominantly high-risk males aged 16 or 17 convicted of serious violent offenses now held in custody (35, 36).

The PBS accurately predicted scores on four related negative outcome CAPS included in the RAI-MH, suggesting that the combined use of the FS and RAI-MH items and calculation of the PBS may have utility as a risk assessment instrument for interpersonal violence and use of coercive interventions even in general mental health care settings, including youth, and in emergency care settings. At the same time, inclusion of the 11 items comprising the PBS on admissions screening instruments in adult and youth correctional settings shows promise for early intervention to prevent negative outcomes even in these settings.

The PBS was fashioned after forensic mental health risk scales, so the fact that individuals with higher PBS scores are more likely to trigger the Harm to Others CAP is no surprise. As we discussed in the introduction, control interventions (restraints, seclusion) are regularly used to control patients who are escalating in threatening and violent behavior, so the fact that participants with higher PBS scores are more likely to trigger the Control Intervention CAP is also not surprising. It also makes sense that the PBS scale predicts the triggering of the Interpersonal Conflict CAP, especially after reviewing the items contained on the instrument. The triggering of the Trauma cap is not so obvious. It is true that the experience of being restrained or secluded may be traumatic. However, there is evidence that these patients have experienced trauma prior to hospitalization. According to Stewart et al., (3) the justice-involved youth in our Youth Sample, compared with both inpatient and outpatient mental health groups, had significantly higher rates of exposure to five potentially traumatic events: parental abandonment, death in the family, failing educational program, being a victim of a crime, and living in a violent community. These findings are consistent with previous literature (37, 38). One of the most oft cited interventions implemented in hospitals to reduce the use of restraints and seclusion is referred to as “Trauma Informed Care (5).” Zarse et al. (39) reviewed a large empirical literature based on data acquired using the Adverse Childhood Experiences Questionnaire. They concluded that exposure to various adverse childhood experiences accumulate in their effects increasing the risk for a wide array of causally interlinked mental illnesses, addictions, and multi-organ medical diseases. Clinical teams, following advice contained in the CAPs, will engage in evidence-based treatments to reduce the factors that lead to these negative outcomes.

## Conclusions

We began this article with a discussion of the two most significant threats facing forensic mental health patients in hospital, namely interpersonal violence, and coercive interventions by staff. We have come to view these two features as a dyad (one thing with two parts; Cambridge English Dictionary). An illustrative scenario will demonstrate what we mean by this.

*A male forensic patient has been recently admitted to the hospital unit. Shortly after, a staff member makes a request or gives a direction, and the patient takes exception. He begins to argue with the staff member and the argument escalates to verbal abuse. Staff begin to worry that the verbal abuse will escalate to violence. Staff attempt de-escalation techniques, but to little effect. At that point in time, the staff have a choice to make; To seclude or not. If they seclude in time, they may prevent a violent outburst and related staff and patient injuries. If they wait, the patient may escalate to violence, at which time seclusion is more difficult to effect*.

So, whether an event such as this is recorded as an incident of violence, or a seclusion depends heavily on decision making by staff. Obviously, this decision making cannot be predicted through an assessment of the forensic patient.

Our research findings, described in this article, suggest that a risk assessment instrument written in interRAI MH format, and based on predominant forensic risk assessment instruments, is predictive of coercive interventions (seclusion in hospital and segregation in corrections). High priority future research should use a longitudinal design to assess predictive validity of the PBS. In addition, future research on the evaluation of risk assessment instruments used to predict negative outcomes in hospital should regard the dependent measure as interpersonal violence and/or coercive interventions. Doing so should increase predictive accuracy of such instruments.

The use of the PBS should never obviate or discourage the use of traditional forensic risk assessments. The VRAG, PCL-R and HCR-20 are currently used by credentialled forensic psychologists or psychiatrists, often in preparation for presentation and cross examination of risk assessment testimony where courts or review boards are considering the liberty interests of an accused person. These assessments require a detailed review of the patient's criminal history, police reports, court, and hospital records. Forensic risk assessments are based on an individual's record of lifetime behavior and experiences. These assessments often take several days to complete, and because of waiting lists and shortages of professional staff, results are often not available to program clinical staff for months. In contrast, the interRAI MH and FS can be completed at intake based on up to 3 days of behavioral observation by psychiatric nursing staff. Therefore, scientifically sound evidence relevant to patient safety might be used to manage the patient's clinical needs immediately. We are not suggesting that the interRAI MH and FS replace traditional risk instruments or become the sole tool for forensic decision-making. However, they can allow for the immediate implementation of preventive care planning and intervention to reduce need for coercive approaches at the beginning of an episode of care. Nevertheless, the traditional instruments are required for formal risk assessments and the communication of a diagnosis (e.g., psychopathy, personality disorder) essential for a comprehensive forensic assessment.

## Data Availability Statement

The datasets presented in this article are not readily available because the youth data are not to be disclosed under any circumstances according to the REB approvals for that data set. Requests to access the datasets should be directed to Howardbarbaree@gmail.com.

## Ethics Statement

The studies involving human participants were reviewed and approved by the REBs at the following institutions: The University of Waterloo, Waypoint Centre for Mental Health Care, the Centre for Addiction and Mental Health, the University of Michigan, the Michigan Public Health Institute, the Michigan Department of Community Health, the University of Toronto, the Centre for Addiction and Mental Health, the University of Western Ontario, and the Ministry of Children and Youth Services (MCYS). Written informed consent from the participants' legal guardian/next of kin was not required to participate in this study in accordance with the national legislation and the institutional requirements.

## Author Contributions

HB was responsible for first drafts of much of the manuscript, overall coordination of author's contributions including data analysis and manuscript drafting and editing. KM was responsible for data collection and analysis of the Forensic Sample and provided comments on earlier drafts. BF was responsible for data collection and analysis of the Correctional Sample, and he took major responsibility for editing and rewriting sections of the manuscript. GB wrote a first draft of the Discussion and provided comments on earlier drafts of the manuscript. SS was responsible for data collection and analysis of the Youth Sample and has provided comments on earlier versions of the manuscript. EH was responsible for data collection and analysis of the Developmental Sample and proofed and edited earlier drafts of the manuscript. JH provided critical comment on the manuscript as a whole and made crucial suggestions regarding the manuscript's Background. All authors contributed to the article and approved the submitted version.

## Funding

Financial support for the work described was provided by the Waypoint Center for Mental Health Care, including financial support for a student (KM). Financial support for the collection of data from the Youth Sample came from a grant from the Canadian Institute of Health Research (CIHR) Partnership for Health Service Improvement, (PHSI), (2013–2020). The research team included: Erickson, P. (Co-PI), Vingilis, E. (Co-PI), SS (Co-PI), Bondy, S., Carlisle, C., Kolla, N., Hamilton, N., and Wheeler, P. *Connecting youth in custody with mental health services*.

## Conflict of Interest

The authors declare that the research was conducted in the absence of any commercial or financial relationships that could be construed as a potential conflict of interest.

## Publisher's Note

All claims expressed in this article are solely those of the authors and do not necessarily represent those of their affiliated organizations, or those of the publisher, the editors and the reviewers. Any product that may be evaluated in this article, or claim that may be made by its manufacturer, is not guaranteed or endorsed by the publisher.
